# Efficacy of Sulphachloropyrazine, Amprolium Hydrochloride, Trimethoprim-Sulphamethoxazole, and Diclazuril against Experimental and Natural Rabbit Coccidiosis

**DOI:** 10.1155/2018/5402469

**Published:** 2018-10-23

**Authors:** Kennedy O. Ogolla, Peter K. Gathumbi, Robert M. Waruiru, Paul O. Okumu, Joyce Chebet, Philip M. Kitala

**Affiliations:** ^1^Department of Veterinary Pathology, Microbiology and Parasitology, University of Nairobi, P.O. Box 29053-00625, Kangemi, Nairobi, Kenya; ^2^Department of Public Health, Pharmacology and Toxicology, University of Nairobi, P.O. Box 29053-00625, Kangemi, Nairobi, Kenya

## Abstract

There are no anticoccidial drugs labelled for rabbits in Kenya and those available are used as extra labels from poultry. The drugs are used in rabbits with limited knowledge of their efficacy and safety. The aim of this study was to determine the efficacy of sulphachloropyrazine, amprolium hydrochloride, and trimethoprim-sulphamethoxazole relative to diclazuril when used curatively against experimental and natural rabbit coccidiosis. In a controlled laboratory trial, sixty (60) rabbits were randomly allocated to six treatment groups, namely, 1A, 2B, 3C, 4D, 5E, and 6F, each with 10 rabbits. Groups 2B, 3C, 4D, 5E, and 6F were experimentally infected with mixed* Eimeria* species while group 1A served as uninfected-untreated (negative) control group. Four of the infected groups were treated with sulphachloropyrazine (5E), amprolium hydrochloride (2B), trimethoprim-sulphamethoxazole (6F), and diclazuril (4D) using dosages recommended by manufacturers. Group 3C served as infected-untreated (positive) control. A field efficacy trial in naturally infected rabbits was then undertaken. The results revealed that sulphachloropyrazine and diclazuril were effective against rabbit clinical coccidiosis by significantly reducing oocyst counts from 149.00±110.39 x 10^4^ to 3.31±0.86 x 10^4^* Eimeria* spp. oocysts per gram of feces (opg) and 59.70±12.35 x 10^4^ to 0.0±0.0 x 10^4^ opg, respectively, in the laboratory trial. Similarly, sulphachloropyrazine and diclazuril recorded reduced oocyst counts in the field trial from 280.33±44.67 x 10^3^ to 0.44±0.14 x 10^3^ opg and 473.44±176.01 x 10^3^ to 0.0±0.0 x 10^3^ opg, respectively. Still, sulphachloropyrazine and diclazuril showed superior efficacy by registering lesion scores and fecal scores close to those of uninfected untreated control group. Trimethoprim-sulphamethoxazole recorded a satisfactory efficacy in the field trial by recording reduced oocyst counts from 266.78±37.03 x 10^3^ to 0.75±0.11 x 10^3^ opg but was not efficacious in the laboratory trial. Amprolium hydrochloride was not efficacious against clinical coccidiosis in both the experimental and field trials.

## 1. Introduction

The most notable of rabbit diseases is coccidiosis which causes massive economic losses in rabbit production [1, 2]. Coccidiosis results in high mortality and morbidity especially among weaner rabbits [2]. Thirteen* Eimeria* species with varied pathogenicity are known to cause coccidiosis in rabbits [1]. Two forms of coccidiosis exist in rabbits (*Oryctolagus cuniculus*): intestinal coccidiosis where the invading protozoan target epithelial cells of different regions of the intestines resulting in moderate to severe damage depending on the virulence of the species [3] and hepatic coccidiosis where the predilection site of* E. stiedae* is the liver [2, 4]. Though most hepatic infections are mild, severe cases can result in progressive emaciation, hepatomegaly with slightly raised yellowish-white nodules, or cords develop which later on tend to coalesce thereby interfering with liver function [4]. The animal presents with polydipsia, icteric membranes, wasting of the back and hind quarters, and enlargement of abdomen [4]. Rabbits with intestinal coccidiosis may present with diarrhoea, dehydration, inappetence, loss of weight, reduced weight gain, rough hair coat, and congested mucous membranes resulting in low productivity [3]. Occurrence of coccidiosis in rabbitries is exacerbated by poor hygiene and high stocking densities which encourage parasite dispersal [5]. Coccidia oocysts have a remarkable ability to survive in exogenous environment making its control by common disinfectants difficult [6]. Currently, several control strategies are used to treat and prevent coccidiosis. Proper hygiene, strict biosecurity, and good husbandry practices have been shown in previous studies to play a significant role in preventing entry and spread of coccidiosis in a rabbitry [1]. Despite their success in the poultry industry, live attenuated and live nonattenuated vaccines produced from precocious lines have been tried with unsatisfactory results in rabbits [7]. Furthermore, natural alternatives extracted from plants, fungus, and microorganisms (prebiotics and probiotics) are currently being used to keep coccidiosis in check [8]. Already published results of the first part of this study revealed that rabbit farmers in Kenya apply the ethno-veterinary use of* Aloe vera* and the nonconventional use of liquid paraffin in the treatment of rabbit coccidiosis with varied efficacies [9]. However, synthetic anticoccidials (both ionophores and synthetic chemicals) remain the mainstream agents used in control of rabbit coccidiosis globally [1]. A study by Peeters et al. [10] demonstrated effectiveness of narasin against mixed hepatic and intestinal coccidiosis. Elsewhere, robenidine, salinomycin, and lerbek have extensively been used in Europe with varied efficacies against hepatic coccidiosis [1, 11]. Similarly, studies have reported varied efficacies following prophylactic and curative use of sulphonamides against coccidiosis [12–15]. Prophylactic and therapeutic use of toltrazuril have recorded good results in countries where they are used [14–17]. In India, amprosol, bifuran, and sulpha-based drugs have been widely used for prevention of rabbit coccidiosis [2]. Prophylactic and curative use of diclazuril against rabbit coccidiosis has recorded impressive results in Italy, France, and Spain [1, 18]. Presently, there are no specific rabbit anticoccidials in Kenya, and farmers use the poultry anticoccidials to treat rabbit coccidiosis. They do this using the poultry dosages with little or no knowledge of their safety and efficacy in rabbits. While resistance has been reported against most of the available anticoccidials [6, 13, 19, 20], no literature exists in Kenya on the efficacies of these anticoccidial agents against local* Eimeria* spp. isolates. Whereas most anticoccidials are indicated for both prophylactic and curative usage, the purpose of this study was to determine the curative (therapeutic) efficacy of sulphachloropyrazine, amprolium hydrochloride, and trimethoprim-sulphamethoxazole and compare them to diclazuril that has proven efficacy elsewhere [18, 20, 21] and has never been used in Kenya.

## 2. Materials and Methods

### 2.1. Experimental Drugs

Three of the most commonly used anticoccidial drugs, sulphachloropyrazine (ESB_3_® manufactured by Novartis AG, Basle, Switzerland), trimethoprim-sulphamethoxazole (Biotrim-Vet® manufactured by Biodeal LTD) and amprolium hydrochloride (Coccid® manufactured by COSMOS LTD) as reported by rabbit farmers in Kenya in a previous baseline survey [9], were selected for experimental trial. Water-soluble sulphachloropyrazine was used as instructed by the manufacturer (2g per liter or 2000ppm). This drug was administered for six days as follows: 1st, 2nd, 3rd, 5th, 7th, and 9th day. Water soluble amprolium hydrochloride 20% was administered at 1g/liter (1000ppm concentration) for 7 consecutive days. Water-soluble trimethoprim-sulphamethoxazole was administered at 1g per liter of water (1000ppm concentration) for 7 days continuously. The above three test drugs were obtained from Nairobi Veterinary Centre, Kenya. Water-soluble diclazuril (Diclosol 1%®) was procured from Pharmaswede Company in Egypt and administered to the rabbits at 10ppm in drinking water for 48 hours continuously.

### 2.2. Experimental Rabbits

A total of 65 weaner (8 weeks to 10 weeks old) rabbits of New Zealand white and California white breeds were obtained from Ngong' National Breeding Centre and used in the experimental phase of this study. The decision to use weaners was based on the fact that this is the most susceptible age group to coccidiosis as demonstrated in previous studies [4, 5, 22, 23]. Fecal samples were collected from the rabbits before and after one-week acclimatization period to confirm they were coccidia-free. Five of the rabbits were used to test the potency of the inoculant. The remaining rabbits were allocated to six treatment groups using a random block design. Anticoccidial-free commercial feed and water were provided to the rabbits* ad libitum*. Basic hygienic measures were maintained throughout the experiment. To prevent cross-contamination, rabbits in the negative control group were housed in the top cages. The study was approved by the Biosafety, Animal Use and Ethics Committee, University of Nairobi, reference number: FVM-BAUEC/2018/144.

### 2.3. Preparation of Inoculant

The inoculant of* Eimeria* oocysts was obtained from the fecal samples of naturally infected rabbits in the field. Ten (10) rabbit farms in Ngong' area which had confirmed clinical coccidiosis were purposively sampled. The samples were processed using a modified McMaster technique with sodium chloride flotation fluid for oocyst detection according to MAFF [24]. The fecal samples were then emulsified in a proportionate amount of flotation fluid (NaCl) and then sieved into 15-liter buckets and basins. To recover oocysts from the flotation fluid, large Petri dishes (150 mm x 25 mm BRAND® Petri dish) were placed afloat on the flotation fluids so that the oocysts could stick on their submerged parts. The Petri dishes were removed after 30 minutes and their submerged parts washed with distilled water into 2,000 ml measuring cylinders which were then topped up with distilled water. Oocysts were recovered through sieving and sedimentation techniques as described by Soulsby [25]. The sporulation of recovered oocysts was done at 27°c in 2.5% potassium dichromate solution for 7 days with 60-80% humidity with on and off aeration by placing water in two standard-size (100mm x 15mm) Petri dishes, a slight modification of a method described by Ryley et al. [26]. The sporulated oocysts were cleared by 5 centrifugation cycles (1500 rpm for 10 minutes each) using distilled water and counted per 1.0 ml using hemocytometry technique. The various* Eimeria* species in the inoculum were then identified based on morphology including size (after measuring 25 oocysts) of each group size as described by [15]. The inoculant dose had* E. perforans *(21%),* E. flavescens *(20%),* E. stiedae *(16%),* E. media *(11.2%),* E. piriformis *(10.6%),* E. intestinalis *(9%),* E. magna *(8%), and* E. coecicola *(4.2%). The inoculant was first tested in 5 pre-trial rabbits at a level of 100,000 mixed oocysts based on previous studies [21, 27–29] which all came down with clinical coccidiosis after 6 to 7 days postchallenge. With potency of the inoculant confirmed, 50 coccidia-free experimental rabbits were orally infected with the inoculant via syringes after overnight starvation

### 2.4. Experimental Design

#### 2.4.1. Laboratory Trials

A total of 60 rabbits were randomly allocated into 6 groups each consisting of 10 rabbits (1A, 2B, 3C, 4D, 5E, and 6F). Groups 1A and 3C served as uninfected untreated (negative control) and infected untreated (positive control), respectively. Rabbits in groups 2B, 3C, 4D, 5E, and 6F were infected with 120,000 sporulated oocysts of mixed* Eimeria* species which were administered orally using a syringe. Treatments were commenced on day 10 when opg counts reached 500,000 and/or when clinical signs of coccidiosis were observed. Groups 2B, 4D, 5E, and 6F were treated with amprolium, diclazuril, sulphachloropyrazine, and trimethoprim-sulphamethoxazole combination, respectively. Faecal samples were collected every other day from the 2nd day postinfection when only a few oocysts were seen to day 30 postinfection. Faecal opg counts of each treatment group were determined by a modified McMaster technique [24]. Daily mortality was recorded and mean weight gain for each group determined at the end of the experiment. In order to assess the lesion score, 3 rabbits from each treatment group were picked randomly for necropsy examination at the end of the experiment in addition to those that died in the course of the experiment. Lesion scores were quantified through macroscopic (gross) examination of the duodenum, jejunum, ileum, caecum, colon, and liver of each rabbit. The lesions were scored as 0 when no evident lesion was seen while a score of 3 was assigned to the severely infected rabbits as was reported by Elbahy [30]. Feces voided were observed and scored from day 1 postinfection. A score of 1 indicating normal well-formed fecal pellets through 5, indicating severe diarrhea with/without profuse amount of blood, was used according to Ramadan [31]. Gall bladder impression smears were prepared routinely and stained with Giemsa.

#### 2.4.2. Field Trials

A total of (10) farms in Kiambu County and Ngong area with confirmed clinical cases of rabbit coccidiosis were recruited for the field trial. Any rabbit with ≥ 200,000 or ≤ 200,000 opg counts but presenting with clinical signs of coccidiosis such as diarrhea, inappetence, and dehydration met the inclusion criteria. The rabbits were then randomly grouped into four treatment groups: F1, F2, F3, and F4. Each treatment group had 90 sick rabbits. Each treatment group was further subdivided into 18 subtreatment groups each containing 5 rabbits giving 18 replications. Group F1 received diclazuril at 10ppm for 48 hrs while group F2 were given sulphachloropyrazine at 2g per liter (2000ppm) on days 1, 2, 3, 5, 7, and 9. Group F3 received trimethoprim-sulphamethoxazole combination at 1g per liter (1000ppm) administered daily for 7 days and finally, group F4 was put under amprolium hydrochloride (20%) treatment at 1g per liter (1000ppm) for 7 consecutive days. Oocyst counts were pooled for each subtreatment group and mean counts determined after every two days up to day 20 posttreatment.

### 2.5. Assessment of Drug Efficacies

Efficacy of the drugs was determined through opg counts, fecal scores, lesion scores, mortality and survival rates, and mean weight gains of the various treatment groups. The effectiveness of the drugs was then determined by comparing the above parameters for the treated groups with those for the positive and negative control groups.

### 2.6. Data Analysis

The data obtained was entered in MS Excel 2016 spreadsheet and cleaned. Analysis of variance was performed by one- or two-way ANOVA as described by GenStat. Significant differences of the means of the different treatment groups were illustrated by Bonferroni multiple comparison tests to control overall significance levels as described in GenStat statistical analysis program (GenStat 15th Edition). The resulting data were presented as mean ± SEM and significance levels stated at p≤0.05.

## 3. Results

### 3.1. Laboratory Trials

#### 3.1.1. Mean Fecal Scores and Standard Error of the Mean (SEM)

Rabbits in the 5 experimentally infected treatment groups presented with clinical signs of loose feces and diarrhea from day 6 postinfection as presented in Table 1. On day 10 postinfection, majority of the rabbits had loose feces while a few had watery diarrhea with/without blood stains. Most of the rabbits in the infected groups showed clinical signs of reduced appetite manifested by feed remaining in the feeders, rough hair coats, distended and pendulous abdomen, dullness, perineal area soiled with feces, marked hepatomegaly on palpation, slight dehydration, and reduced weight. There was a significant difference (p<0.05) in fecal scores between the infected groups and the uninfected group from day 6 postinfection.

Treatments with diclazuril (4D) and sulphachloropyrazine (5E) showed satisfactory efficacy from day 9 posttreatment through alleviation of diarrhea and production of normal fecal pellets as shown in Table 2. Furthermore, 4D and 5E treatment groups revealed a significant (p<0.05) improvement in fecal score from 2.67±0.21 for the two treatment groups to 1.17±0.17 and 1.33±0.21, respectively, compared to that of the positive control group (3C) that recorded a slight reduction from 3.17±0.31 to 3.0±0.32. Group 4D recorded a fecal score even better than that of the negative control group (1A) of 1.33±0.21 at the end of the experiment. There was no significant difference (p>0.05) in fecal scores between amprolium (2B) and trimethoprim-sulphamethoxazole (6F) treatment groups relative to the positive control group.

#### 3.1.2. Oocyst Shedding before and after Treatment

Oocyst counts in all the treatment groups ranged from 0 to <1.0 x 10^3^/g on infection day (day 0). There was no significant difference (p>0.05) in oocyst counts between infected groups and the uninfected negative control group (1A) from day 0 to day 4 postinoculation (Table 3). However, from day 6 postinoculation onwards, there was a rapid increase in oocyst shedding in the infected groups compared to group 1A which peaked on days 7 and 12 postinfection. On the other hand, the positive control group (3C) demonstrated a steady increase in oocysts shed up to day 20 postinfection after which the numbers started to decrease.

**Figure 1:**
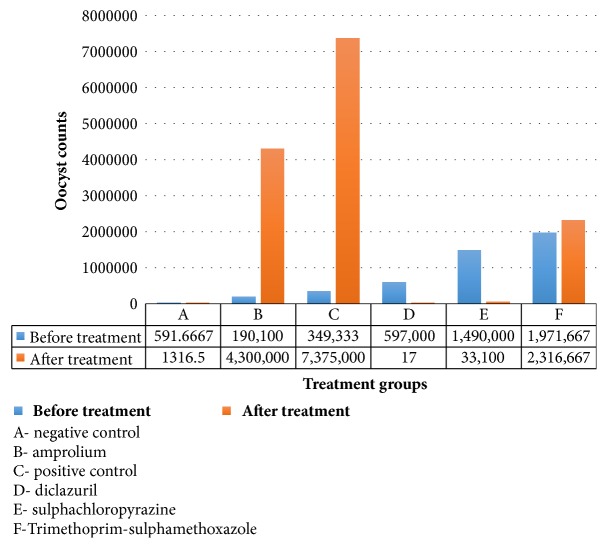
A bar graph showing reduction in oocysts shed before and after treatment in rabbits on drug efficacy study for experimental coccidiosis.

Treatment groups 4D and 5E had a significant (p<0.05) reduction in mean oocysts shed on day 7 posttreatment compared to infected untreated group (3C) (Table 4). On day 13 posttreatment, group 4D recorded 0.00±0.00 oocyst count impressively better than even that of negative control group 0.173±0.068 x 10^4^/g (3 logarithms' difference lower) while group 5E recorded an oocyst count of 2.03±0.829 x 10^4^/g (about 1 logarithm higher than group 1A). At the termination of the experiment (day 20 posttreatment), the mean number of oocysts shed remained extremely low in groups 4D and 5E compared to groups 3C, 2B, and 6F (about 5 and 2 logarithms' difference higher, respectively, for all the groups) as illustrated in Table 4. Group 6F recorded a higher reduction in oocysts shed on day 7 posttreatment 61.17±10.603 x 10^4^/g compared to groups 2B and 3C. However, the mean oocysts shed by group 6F started to rise again from day 13 and by 20 days posttreatment they had reached 231.67±51.43x10^4^/g. However, this was still significantly reduced (p<0.05) relative to 737.50±213.478 x 10^4^/g of group 3C. On the other hand, mean oocyst count shed by group 2B on day 7 posttreatment was higher, 357.67±123.451 x 10^4^/g, relative to 170.20±68.921 x 10^4^/g of group 3C though not significantly different (p>0.05). In this study, amprolium (group 2B) had the least efficacy. The exact oocyst counts before and after treatment following experimental infection are shown in Figure 1.

#### 3.1.3. Total Mean Macroscopic Lesion Scores

Diclazuril was highly efficacious (p<0.05) in the reduction of the hepatic and intestinal lesion scores (0.33±0.33) compared to positive control group 3C (2.67±0.33) with a lesion score difference of more than 2. Though significantly efficacious (p<0.05) compared to group 3C, group 5E (1.33±0.33) had some mild lesions compared to group 2B as depicted in Table 5. Strikingly, there was no significant difference (p>0.05) in lesion scores recorded for groups 2B, 6F, and 3D.

**Figure 2:**
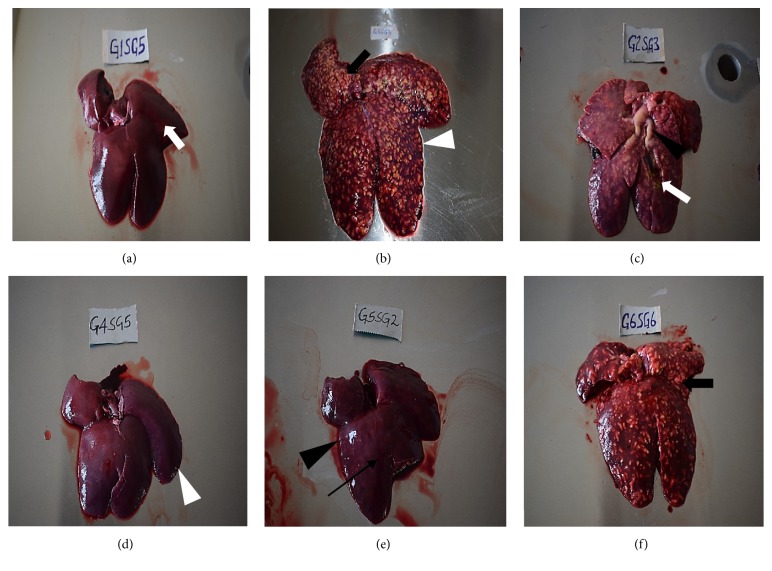
Hepatic lesions at termination of the drug efficacy study in experimentally induced coccidiosis infection. (a) Normal liver with normal architecture of incised section (white arrow) from negative control group, (b) liver with hepatomegaly manifested by diminished sharp edges (white arrow head) with raised multinodular lesions due to hepatic coccidiosis from positive control group, (c) markedly enlarged liver with multinodular whitish-yellow lesions and distended bile duct (arrow head), incised section with greenish-yellow material (white arrow) from amprolium treatment group, (d) liver from diclazuril treatment group without any significant lesion, note the sharp edges (white arrow head), (e) slightly enlarged liver (loss of sharp edges, black arrow head) with tiny whitish-yellow fibrotic spots after healing (black arrow) from sulphachloropyrazine group, and (f) enlarged liver with raised multinodular whitish-yellow lesions from trimethoprim-sulphamethoxazole group.

There was evident congestion, hepatomegaly (almost 3 times the normal size), and increased dark straw coloured peritoneal fluid in groups 6F, 2B, and 3C. Additionally, livers from the three treatment groups had raised yellowish-white multinodular lesions 1-2 cm in diameter covering the entire liver surface and its parenchyma. The gallbladder was markedly distended and contained thick yellowish-white contents whose consistency ranged from free-flowing greenish content to firm cheesy material. There were fibrin strands on the surfaces of the livers with numerous necrotic foci. On incision, the liver parenchyma from these treatment groups was firmer compared to those of negative control group that had a soft consistency. Group 5E had mild to moderate hepatomegaly (between half to twice normal size), slightly raised nodular lesions (1mm–1 cm in diameter) with mostly white contents, and slightly-moderately distended gallbladder with greenish yellow contents. Livers from the group (4D) treated with diclazuril did not present with significant gross lesions relative to group 1A apart from the few fibrotic areas (Figure 2).

**Figure 3:**
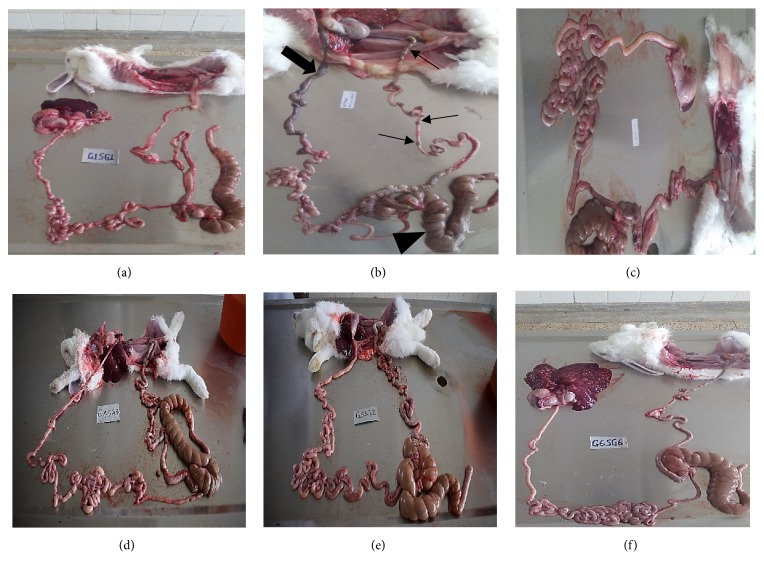
Intestinal lesions from the various treatment groups at termination of the drug efficacy study on experimentally induced coccidiosis infection. (a) Negative control group, (b) intestines from a positive control group rabbit showing marked congestion in the duodenal section (arrow) and in the caecum (arrow head) with well-formed fecal pellets in the colon (thin arrows), (c) amprolium group, (d) diclazuril group, (e) sulphachloropyrazine group, and (f) trimethoprim-sulphamethoxazole group.

Macroscopic intestinal lesions were relatively less severe in comparison to the hepatic lesions. The intestinal lesions ranged from severe congestion, mild haemorrhages in the lumen, hyperemia of the intestinal mucosa, ballooning of caecum, and edema of intestinal mucosa in groups 2B, 3C, and 6F to fairly normal intestines in 1A, 4D, and 5E treatment groups (Figure 3). The raised nodular lesions observed in the liver were absent in the intestines.

#### 3.1.4. Liver Impression Smears

**Figure 4:**
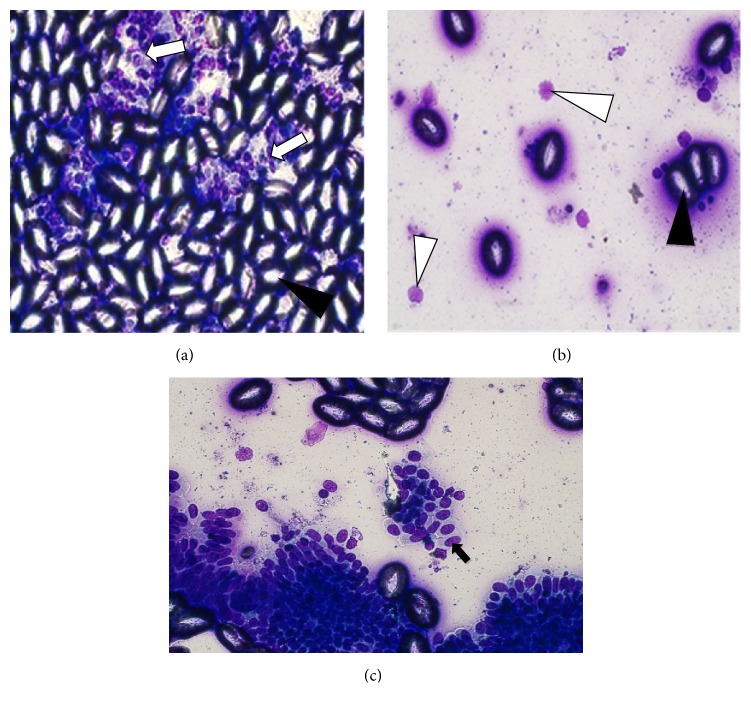
Impression smear characteristics of the liver on drug efficacy study in experimentally induced coccidiosis infection. (a) Clear, oval to elliptical-shaped fully formed oocysts (black arrow head) and hepatobiliary parenchymal cells (white arrow) from amprolium group at x400, (b) macrogametocytes (white arrow head) and few fully formed oocysts (black arrow head) from sulphachloropyrazine group at x400, and (c) cluster of biliary epithelial cells containing numerous microgametocytes from trimethoprim-sulphamethoxazole group (black arrow).

The liver impression smears from treatment groups 2B, 3C, and 6F had numerous clear fully formed coccidial oocysts mixed with few hepatobiliary parenchymal cells (Figure 4). Ellipsoidal fully formed nonsporulated oocyst was the predominant developmental stage from the smears. The oocysts had a smooth, pink wall and a flat micropyle. Immature developmental stages including small microgametocytes of varied shapes within epithelial cells of the ducts (Figure 4(c)) and round macrogametocytes filled with uniform bluish-pink cytoplasmic granules (Figure 4(b)) were present in impression smears from 2B, 3C, and 6F treatment groups. Numerous clusters of cuboidal-columnar epithelial cells of the bile ducts and few inflammatory cells were also seen in these treatment groups. On the other hand, impression smears from sulphachloropyrazine (5E) treatment group had comparatively fewer oocysts compared with the three groups. However, smears from diclazuril and negative control groups were negative for oocysts. These results indicate that diclazuril completely eliminated hepatic coccidiosis while sulphachloropyrazine had more than average efficacy against hepatic coccidiosis.

#### 3.1.5. Survival Percentages, Average Weights, and Weight Gains/Loss

The highest mortality rate (60%) attributed to coccidiosis was recorded by groups 2B, 3C (50%), and 6F (40%). The lowest mortality rate of 20% was recorded in groups 5E and 4D. Rabbits recruited for this study all had weights around 820g at the beginning of the study. Group 1A had the highest (p<0.05) mean weight gain (38%) at termination of the study. Groups 4D (17%) and 5E (12.35%) also recorded significantly (p<0.05) increased weight gains. Group 6F recorded the highest mean weight loss of 13.17% followed by groups 2B (3.7%) and 3C (1.21%), respectively. The mean weight gain in the six treatment groups was significantly different at p<0.05.

### 3.2. Field Trial

Table 6 summarizes the effects of respective treatments on oocyst shedding (as an indicator of efficacy) with time. In this field trial, diclazuril and sulphachloropyrazine were efficacious against coccidiosis as indicated by reduction in oocysts shed on day of treatment relative to day 16 posttreatment (Table 6). Trimethoprim-sulphamethoxazole combination had moderate to satisfactory efficacy while amprolium hydrochloride was not able to control clinical coccidiosis in the field as indicated by the high oocyst counts at trial termination (Table 6).

## 4. Discussion

In the randomized controlled experimental trial, clinical signs of watery diarrhea with/without blood stains, loose feces, reduced appetite, rough hair coat, distended and pendulous abdomen, dullness, reduced weight, soiled perineal area, hepatomegaly on palpation, and slight dehydration were observed. This is in concurrence with an earlier experimentally induced coccidiosis study [29] and other studies for hepatic [2, 22] and intestinal coccidiosis [23, 32]. In agreement with Al-Naimi [22], jaundice was only seen in very severe cases. The liver impression smears of hepatic coccidiosis described in this study were as described in other works [3, 33]. Our study demonstrated the superior efficacy of curative use of diclazuril and sulphachloropyrazine against rabbit coccidiosis. Similar efficacies following curative use of diclazuril in resolving clinical signs of coccidiosis have been reported in other studies [18, 21] even against* Eimeria* spp. resistant to other anticoccidial drugs [20]. Efficacy of subcutaneously administred diclazuril against* Eimeria* infection was demonstrated by Pan et al. [34]. Furthermore, the superior efficacy of diclazuril in elimination of oocysts shed has been reported in several rabbit studies [13, 18, 21, 35]. Effectiveness of sulphachloropyrazine when used curatively against clinical coccidiosis reported in the present study agrees with an earlier study by Kolabskii et al. [36]. Similarly, sulphachloropyrazine has been shown to be effective against poultry* Eimeria* spp. [37, 38]. On the other hand, trimethoprim-sulphamethoxazole recorded moderate efficacy in the field trial but was not effective against coccidiosis in the controlled laboratory trial. The moderate efficacy in the field trial may be attributed to the low intensity of infections during the trial. Amprolium, when used therapeutically, was not effective against rabbit coccidiosis in both laboratory and field trials. These results agree with an earlier study by Laha [39] that demonstrated the inability of amprolium to reverse active coccidial infection in rabbits and less than satisfactory efficacy in broiler chickens as reported by Das [38]. In a recent efficacy study from Ethiopia, Hunduma and Kebede [40] reported that amprolium was ineffective in controlling coccidiosis of poultry. The ineffectiveness of amprolium in our study may be attributed to the development of resistance that may have arisen over the years following its indiscriminate use and misuse by the farmers as was established in our already published baseline survey [9]. Efficacy of amprolium has been reported to be region specific depending on how the drug has been used in such regions over time which may or may not have led to development of resistance [41]. Nonetheless, better efficacies have been reported following prophylactic use of amprolium against intestinal coccidiosis [15, 16, 41], and when used concurrently with other anticoccidials. Since, however, the present study tested the curative efficacy of these anticoccidials against clinical coccidiosis, the authors note that the efficacies may differ when they are used prophylactically or in early stages of infection before the establishment of a clinical disease.

## 5. Conclusions

Diclazuril and sulphachloropyrazine were efficacious against rabbit coccidiosis. Trimethoprim-s*ulphame*thoxazole was not able to control coccidiosis infection at the recommended poultry reference dosages in the controlled experimental trials. However, its efficacy was moderate to satisfactory in the field trial at the recommended dosages. On the other hand, amprolium was not efficacious against intestinal and hepatic coccidiosis in both the controlled laboratory and field trials. The study recommends training of farmers and field extension officers on the prudent use of available efficacious anticoccidials and best rabbit management practices to promote rabbit production in Kenya.

## Figures and Tables

**Table 1:** Mean fecal scores from the day of inoculation to day 11 postinfection on drug efficacy study in experimentally induced coccidiosis infection.

**Group**	**Inoculation day (Day 0)**	**Day 7**	**Day 10**	**Treatment day ** **(Day 11)**
Negative control (1A)	1.0±0.00	1.0±0.00^a^	1.17±0.17^a^	1.33±0.21^a^

Amprolium (2B)	1.0±0.00	2.0±0.37^ab^	2.83±0.31^b^	3.0±0.26^b^

Positive control (3C)	1.0±0.00	2.17±0.31^ab^	3.0±0.26^b^	3.17±0.31^b^

Diclazuril (4D)	1.0±0.00	2.33±0.33^b^	2.5±0.34^b^	2.67±0.21^b^

Sulphachloropyrazine (5E)	1.17±0.17	2.17±0.31^ab^	2.67±0.21^b^	2.67±0.21^b^

Trimethoprim-sulphamethoxazole (6F)	1.0±0.00	2.67±0.21^b^	2.67±0.33^b^	2.83±0.31^b^

SD	0.167	0.826	0.878	0.838

**P-value**	**0.435**	**0.007**	**<0.001**	**<0.001**

Values with different superscripts in a column are significantly different at p<0.05.

Fecal score was done according to Ramadan [22] where a score of 1 indicated normal well-formed pellets through 5, indicating severe diarrhea with/without profuse amount of blood.

**Table 2:** Mean fecal scores from the day of treatment to day 20 posttreatment on drug efficacy study in experimentally induced coccidiosis infection.

**Groups**	**Treatment day**	**Day 9**	**Day 17**	**Day 20**
Negative control (1A)	1.33±0.21^a^	1.33±0.21^a^	1.17±0.19^a^	1.17±0.18^a^

Amprolium (2B)	3.0±0.26^b^	3.17±0.40^b^	2.50±0.24^b^	2.25±0.23^b^

Positive control (3C)	3.17±0.31^b^	3.0±0.32^b^	2.75±0.24^b^	3.0±0.00^b^

Diclazuril (4D)	2.67±0.21^b^	1.17±0.17^a^	1.17±0.19^a^	1.0±0.18^a^

Sulphachloropyrazine (5E)	2.67±0.21^b^	1.33±0.21^a^	1.20±0.21^a^	1.0±0.20^a^

Trimethoprim-sulphamethoxazole (6F)	2.83±0.31^b^	2.0±0.37^ab^	2.67±0.19^b^	2.33±0.18^b^

**SD**	0.838	1.043	0.860	0.844

**p-value**	**<0.001**	**<0.001**	**<0.001**	**<0.001**

Values with different superscripts in a column are significantly different at p<0.05.

Fecal score was done according to [22] where a score of 1 indicated normal well-formed pellets through 5, indicating severe diarrhea with/without profuse amount of blood.

**Table 3:** Oocyst counts from the day of inoculation to day 10 postinfection on drug efficacy study in experimentally induced coccidiosis infection.

	**Oocysts shed per treatment group x 10** ^**4**^ ** per gram of feces**				
**Group**	Inoculation (Day 0)	Day 4	Day 6	Day 8	Day 10
Negative control (1A)	0.01±0.01	0.03±0.02	0.02±0.00	0.02±0.00	0.06±0.023^a^

Amprolium (2B)	0.01±0.00	0.09±0.02	3.80±0.87	13.97±7.33	19.01±9.57^ab^

Positive control (3C)	0.01±0.01	0.25±0.02	3.82±1.47	15.63±8.79	34.93±16.28^ab^

Diclazuril (4D)	0.01±0.01	0.34±0.15	11.44±3.54	28.22±9.38	59.70±12.35^ab^

Sulphachloropyrazine (5E)	0.01±0.01	0.66±0.48	12.40±9.54	56.97±38.69	149.00±110.39^ab^

Trimethoprim-sulphamethoxazole (6F)	0.01±0.01	0.26±0.06	8.00±4.28	26.13±12.13	197.17±92.66^b^

**p-value**	**0.93**	**0.34**	**0.36**	**0.34**	**0.15**

Values with different superscripts in a column are significantly different at 0.05.

**Table 4:** Oocyst counts on the day of treatment to day 20 posttreatment on drug efficacy study in experimentally induced coccidiosis infection.

**Mean oocyst shed per treatment group x 10** ^**4**^ **/gram of feces (Days posttreatment)**					
Group	Treatment day	Day 7	Day 13	Day 17	Day 20
Negative control (1A)	0.06±0.02^a^	0.09±0.03^a^	0.173±0.07^a^	0.14±0.04^a^	0.14±0.04^a^

Amprolium (2B)	19.01±9.57^ab^	357.67±123.45^b^	416.83±129.86^a^	429.60±129.85^ab^	430.00±62.45^ab^

Positive control (3C)	34.93±16.28^a^	170.20±68.92^ab^	432.40±142.79^a^	642.40±177.50^b^	590.02±96.13^b^

Diclazuril (4D)	59.70±12.35^ab^	0.12±0.10^a^	0.00±0.00^a^	0.00±0.00^a^	0.00±0.00^a^

Sulphachloropyrazine (5E)	149.00±110.39^ab^	0.83±0.40^a^	2.03±0.83^a^	2.03±0.70^a^	3.31±0.86^a^

Trimethoprim-sulphamethoxazole (6F)	197.17±92.66^b^	61.17±10.60^a^	230.50±154.30^a^	358.00±163.17^ab^	231.67±51.4^a^

**p-value**	**0.154**	**<0.001**	**0.008**	**<0.001**	**<0.001**

Values with different superscripts in a column are significantly different at 0.05.

**Table 5:** Total mean lesion scores in the six treatment groups on drug efficacy study in experimentally induced coccidiosis infection.

**Group**	**Animal 1**	**Animal 2**	**Animal 3**	**Mean**
Negative control (1A)	0.00	0.00	0.00	0.00^a^

Amprolium (2B)	3.00	2.00	3.00	2.67^b^

Positive control (3C)	2.00	3.00	3.00	2.67^b^

Diclazuril (4D)	1.00	0.00	0.00	0.33^a^

Sulphachloropyrazine (5E)	2.00	1.00	1.00	1.00^ab^

trimethoprim-sulphamethoxazole (6F)	3.00	3.00	2.00	2.67^b^

Values with different superscripts in a column are significantly different at 0.05.

**Table 6:** Oocysts shed from the day of treatment (day 0) to day 20 posttreatment on drug efficacy study in natural coccidiosis infection.

	**Oocysts shed per treatment group x 10** ^**3**^ ** per gram of feces**				
**Treatment group**	1st day of treatment (Day 0)	Day 6	D ay 10	Day 16	Day 20
Diclazuril (F1)	473.44±176.01	1.13±0.73^a^	0.13±0.10^a^	0.04±0.03^a^	0.00±0.00^a^

Sulphachloropyrazine (F2)	280.33±44.67	15.54±3.96^a^	1.07±0.22^a^	0.59±0.14^a^	0.44±0.14^a^

Trimethoprim-sulphamethoxazole (F3)	266.78±37.03	40.34±9.80^a^	1.36±0.31^a^	0.75±0.11^a^	0.91±0.11^a^

Amprolium (F4)	454.06±93.93	318.43±72.94^b^	188.31±45.86^b^	232.47±61.97^b^	258.92±70.15^b^

**p-value**	**0.345**	**<0.001**	**<0.001**	**<0.001**	**<0.001**

Values without similar superscript in a column are significantly different at 0.05.

## Data Availability

Data on this experiment can be accessed from https://data.mendeley.com/library.

## Supplementary Materials

Supplementary Materials (1) Gross lesion scoring criteria used to quantify the lesions in the laboratory trial. (2) Liver impression smears from the six treatment groups showing varying number of oocysts. A1, negative control; 2B, amprolium; 3C, positive control; 4D, diclazuril; 5E, sulphachloropyrazine; and 6F, trimethoprim-sulphamethoxazole. All the slides were stained using Giemsa stain. Note that only the background is stained as the oocysts do not take the stain. (3) Gross hepatic lesions seen in the six treatment groups; 3C, positive control group; 1A, negative control group; and 2B, amprolium group. Note the hepatic multinodular lesions (arrow) and the markedly distended bile duct (arrow head).
