# Supplementary material for: Efficacy of Sulphachloropyrazine, Amprolium Hydrochloride, Trimethoprim-Sulphamethoxazole, and Diclazuril against Experimental and Natural Rabbit Coccidiosis
Source: J Vet Med. 2018 Oct 23;2018:5402469. doi: 10.1155/2018/5402469 (PMC6218750; doi:10.1155/2018/5402469)
Supplement: Supplementary Materials — (1) Gross lesion scoring criteria used to quantify the lesions in the laboratory trial. (2) Liver impression smears from the six treatment groups showing varying number of oocysts. A1, negative control; 2B, amprolium; 3C, positive control; 4D, diclazuril; 5E, sulphachloropyrazine; and 6F, trimethoprim-sulphamethoxazole. All the slides were stained using Giemsa stain. Note that only the background is stained as the oocysts do not take the stain. (3) Gross hepatic lesions seen in the six treatment groups; 3C, positive control group; 1A, negative control group; and 2B, amprolium group. Note the hepatic multinodular lesions (arrow) and the markedly distended bile duct (arrow head). [file 5402469.f1.docx]

**Supplementary materials**

1. Lesion scoring criteria used in the study

| **Grade** | **Lesion description** |
| --- | --- |
| Grade 0(-)Gr | No evident lesions |
| Grade 1(+) | Slight hyperemia of the intestinal wall, mild thickening of the intestinal wall and 1-3 focal lesions in a length of 3cm of the intestinal wall, slight hepatomegaly (increased by half the normal size) and 1-5 less than 1cm nodular lesions on the liver |
| Grade 2(++) | Moderate hyperemia of the intestinal wall, mild thickening of the intestinal wall, 3-6 focal lesions in 3cm length of the intestinal wall, ballooning of the caecum, moderate hepatomegaly (twice normal size), 6-11 raised nodular lesions 1cm in size on the liver |
| Grade 3(+++) | Severe congestion of the intestinal wall, increased thickening of the intestinal wall, ballooning of the caecum and presence of bloody caecal core, marked hepatomegaly (more than twice normal size, more than 11 raised nodular lesions 1-2 cm in size on the liver |

1. Liver impression smears from the six treatment groups

| 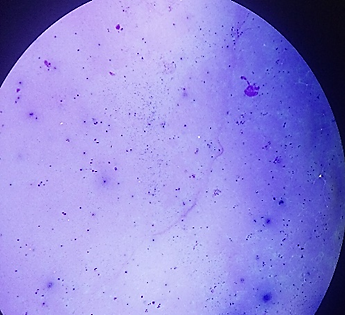  **1A** | 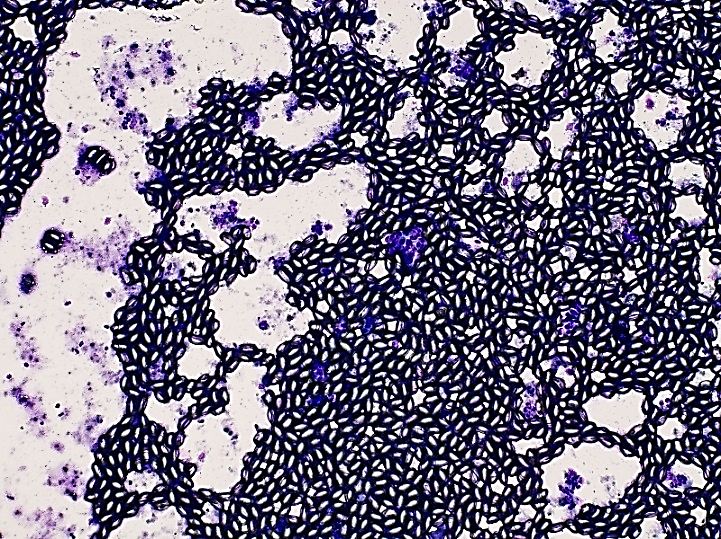  **2B** |
| --- | --- |
| 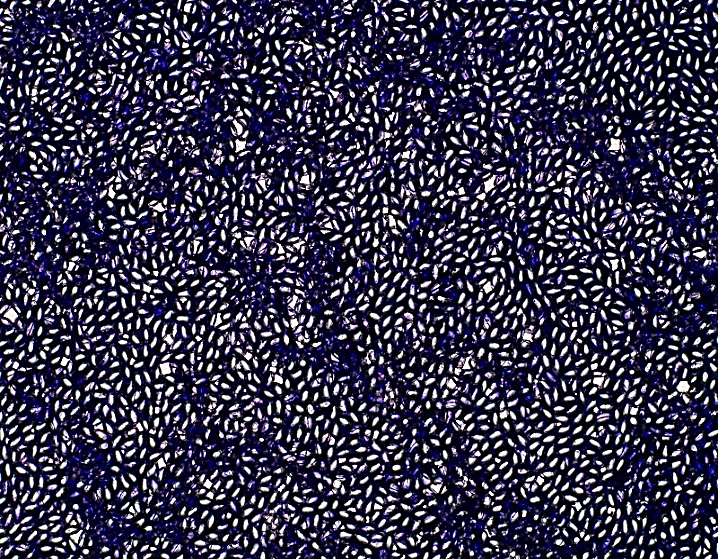  **3C** | 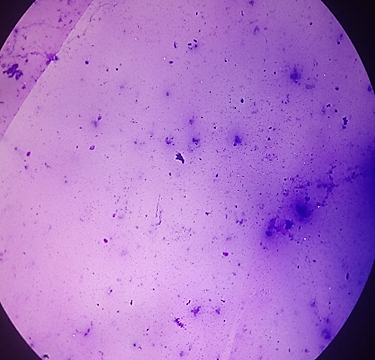  **4D** |
| 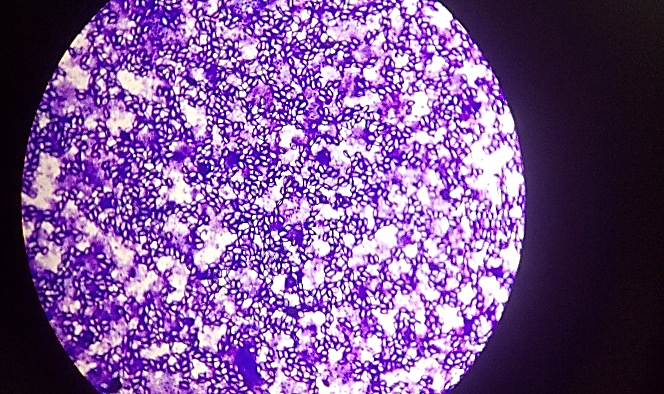  **5E** | 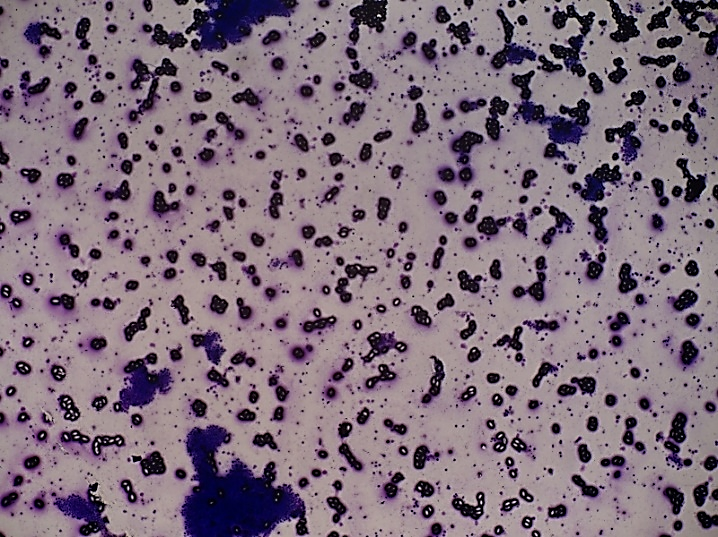  **6F** |

Figure showing liver impression smears from the six treatment groups at x40. The oocysts counts did not vary much in the animals sacrificed per treatment group. A1- Negative control, 2B- Amprolium, 3C- Positive control, 4D- Diclazuril, 5E- Sulphachloropyrazine and 6F – Trimethoprim-sulphamethoxazole. All the slides were stained by Giemsa stain. Note that only the background is stained as the oocysts do not take the stain.

1. Gross hepatic lesions from the six treatment groups

| 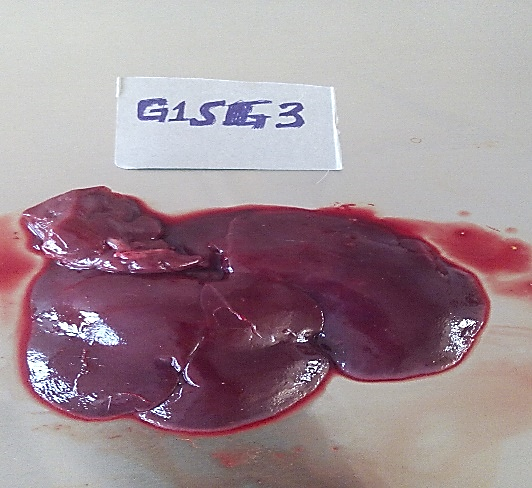  1A | 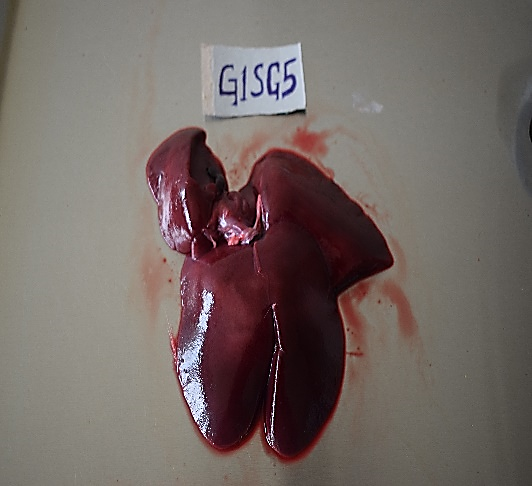  1A | 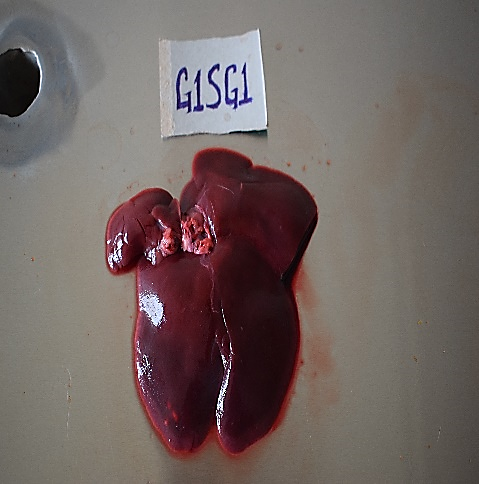  1A |
| --- | --- | --- |
| 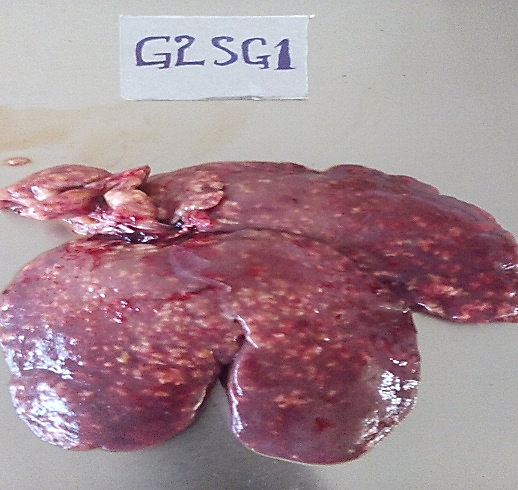2B | 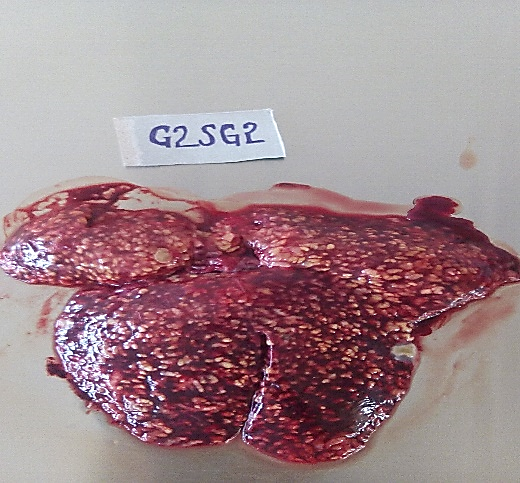  2B | 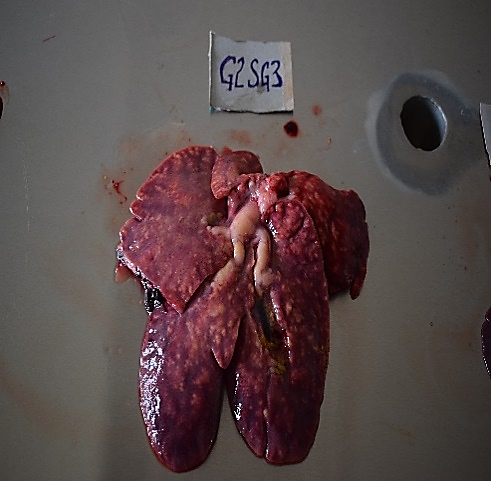  2B |
| 3C  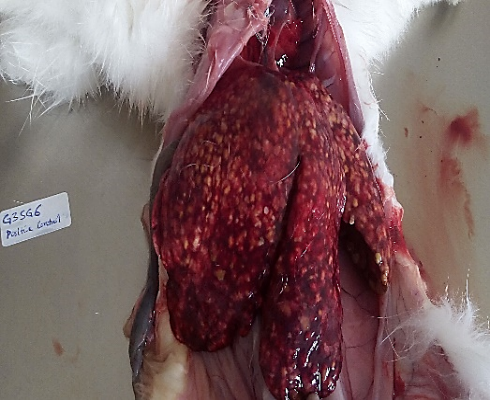 | 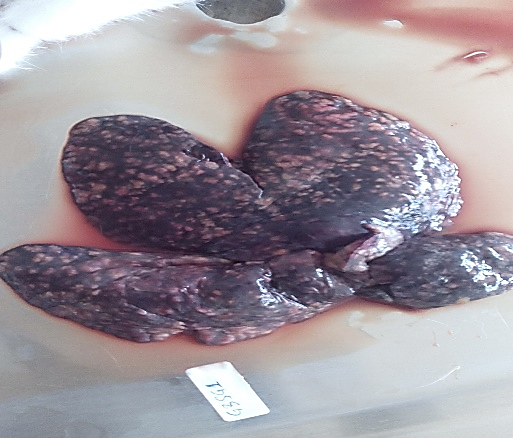  3C | 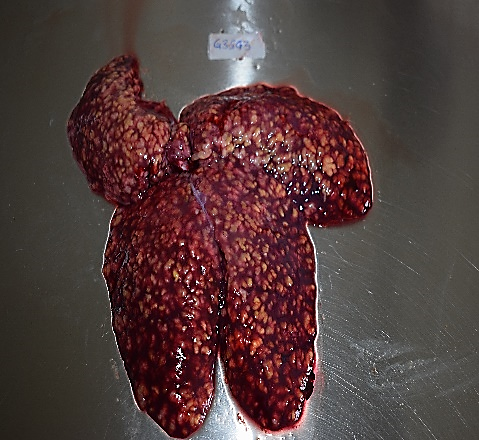  3C |
| 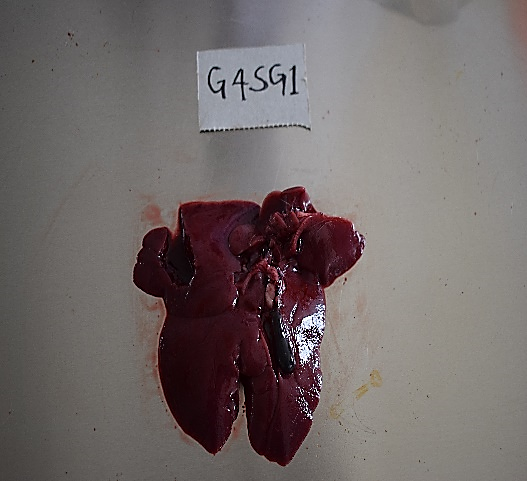4D | 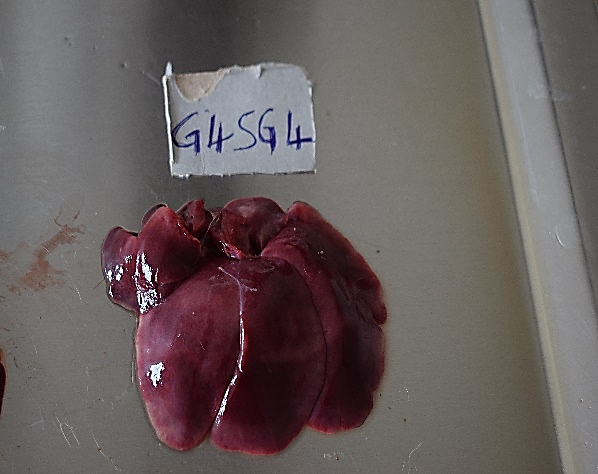4D | 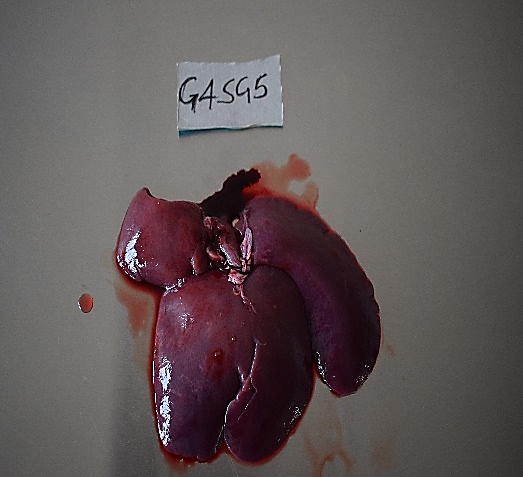 4D |
| 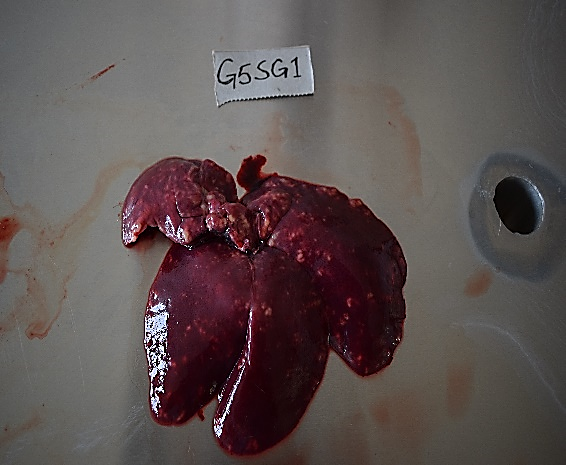  5E | 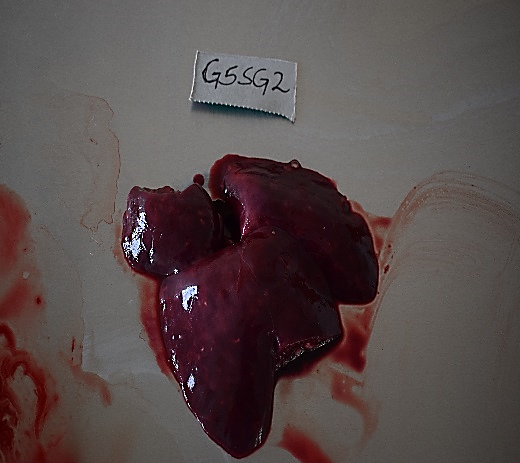  5E | 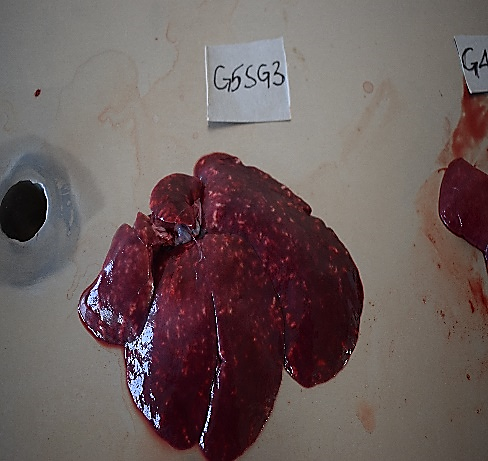  5E |
| 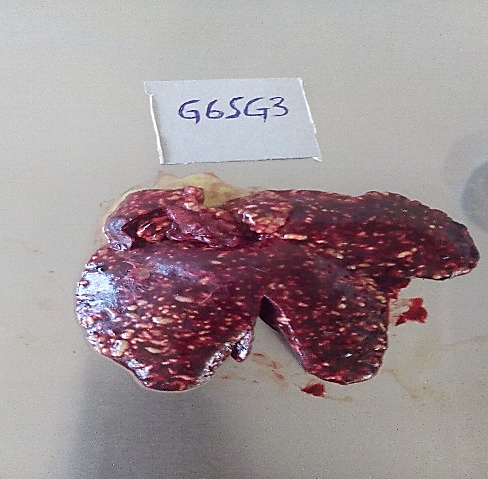  6F | 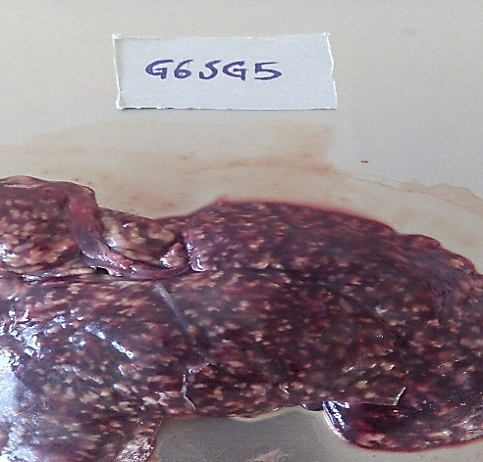  6F | 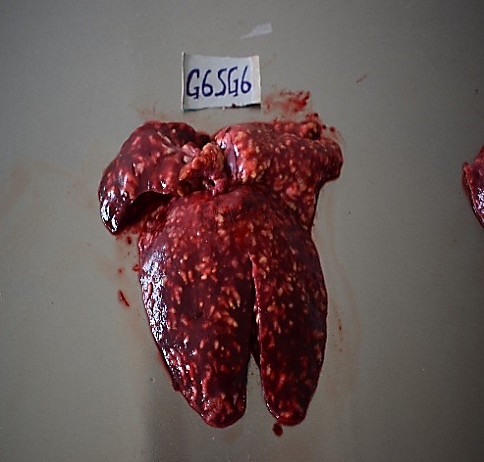  6F |

Figure 2: Hepatic lesions at the termination of the experiment. 1A- normal liver with normative architecture of incised section (white arrow) from negative control group, 2B-markedly enlarged livers from amprolium treatment group with multinodular whitish-yellow lesions and distended bile duct (Arrow head), 3C-livers with hepatomegaly manifested by diminished sharp edges (white arrow head) with raised multinodular lesions due to hepatic coccidiosis from positive control group, 4D- liver from diclazuril treatment group without any significant lesion, note the sharp edges (white arrow head), 5E-slightly enlarged liver (loss of sharp edges –black arrow head) with tiny whitish-yellow fibrotic spots after healing from sulphachloropyrazine group (thin arrow), 6F-enlarged liver with raised multinodular whitish-yellow lesions from trimethoprim-sulphamethoxazole group.
